# High-Stringency Evaluation of the Automated BD Phoenix CPO Detect and Rapidec Carba NP Tests for Detection and Classification of Carbapenemases

**DOI:** 10.1128/JCM.01215-17

**Published:** 2017-11-27

**Authors:** Gina Thomson, David Turner, William Brasso, Susan Kircher, Thierry Guillet, Kenneth Thomson

**Affiliations:** a University of Louisville Hospital, Louisville, Kentucky, USA; b University of Louisville School of Medicine, Louisville, Kentucky, USA; c BD Diagnostics Systems, Sparks, Maryland, USA; Marquette University

**Keywords:** CPO, CPO Detect, KPC, NDM, Rapidec Carba NP, VIM, carbapenemase

## Abstract

There is an urgent need for rapid, accurate detection and classification of carbapenemases. The current study evaluated the automated BD Phoenix CPO Detect and the manual bioMérieux Rapidec Carba NP tests for meeting these needs. Both tests were challenged with 294 isolates of Enterobacteriaceae spp., Pseudomonas aeruginosa, and Acinetobacter baumannii chosen to provide extreme diagnostic difficulty. Carbapenemases such as KPC, NMC-A, IMI, SME, NDM, SPM, IMP, VIM, and OXA-23, 40, 48, 58, 72, 181, and 232 were produced by 243 isolates and 51 carbapenemase-negative isolates included porin mutants and producers of extended-spectrum β-lactamases (ESBLs), AmpCs, K1, and broad-spectrum β-lactamases. Both tests exhibited high sensitivity of carbapenemase detection (>97%). Due to the highly challenging carbapenemase-negative isolates, specificities were lower than typical for evaluations involving mostly routine clinical isolates. BD Phoenix CPO Detect was 68.6% specific and Rapidec Carba NP was 60.8% to 78.4% specific, depending on how borderline results were interpreted. Only BD Phoenix CPO Detect classified carbapenemases. It correctly classified 85.0% of class A, 72.4% of class B, and 88.6% of class D carbapenemases. Importantly with respect to empirical therapy with new β-lactamase inhibitor combinations such as ceftazidime/avibactam, no class B carbapenemases were misclassified as class A carbapenemases. Both tests offer advantages. Used alone, without initial susceptibility tests, Rapidec Carba NP can provide positive results for some isolates after only 10 to 30 min incubation. BD Phoenix CPO Detect provides novel advantages such as automated carbapenemase detection, inclusion in susceptibility panels to eliminate delays and subjectivity in initiating carbapenemase tests, and classification of most carbapenemases.

## INTRODUCTION

Carbapenemase-producing organisms (CPOs) are Gram-negative bacteria that possess a transmissible carbapenemase and are typically resistant to most (sometimes all) antibiotics, leaving few to no therapeutic options (1). The first CPO, a strain of Pseudomonas aeruginosa isolated in Japan, was reported in 1991 (1, 2). Since then, CPOs have spread to produce a global pandemic of high mortality that is sometimes reported to be similar to Ebola (3). This pandemic is the consequence of ineffective infection control, failures to provide timely, effective therapy for infected patients and, for over a decade, failure to recognize the enormity of the CPO threat. Therapeutic outcome data indicate that CPO infections differ from other Gram-negative infections in that monotherapy with a single active drug (to which pathogen is susceptible *in vitro*) is essentially the same as no active therapy. In contrast, combination therapy with at least two active agents reduces mortality, particularly if the combination includes a carbapenem (4). This means that *in vitro* susceptibility results on their own may provide a clinically misleading guide to therapy through failing to indicate the need for combination therapy. This concern is supported by a recent report of a large global surveillance study in which approximately 10% of CPOs were imipenem-susceptible (5). This suggests that laboratories that do not perform carbapenemase tests on carbapenem-susceptible isolates may be placing patients at increased risk of therapeutic failures. Combination therapy may also prevent the emergence of total antibiotic resistance during therapy (6–10). This is an important consideration because the clinician may have only one opportunity to deliver effective therapy and the wrong choice could doom the patient to die from an untreatable infection (1). New β-lactamase inhibitor combinations, such as ceftazidime/avibactam, have the potential to reduce mortality due to class A CPO infections. The future utility of such agents is threatened by intrinsically resistant class B CPOs (11), the emergence of resistance during therapy of infections by KPC-producers (12, 13), and CPOs that produce multiple carbapenemases (14). These threats make it essential to minimize the selection of resistance by avoiding unnecessary use of these agents. Accurate identification of the molecular classes of carbapenemases can help to optimize use of the new β-lactamase inhibitor combinations. Currently marketed rapid phenotypic CPO tests do not classify carbapenemases. Similar to the Phoenix ESBL test, the BD Phoenix CPO Detect (BD Diagnostics Systems, Sparks, MD) is an automated investigational test designed for inclusion in all routine Gram-negative susceptibility panels to detect and classify carbapenemases. It utilizes nine test wells on the Phoenix panel, each containing a β-lactam antibiotic, alone or in combination with various β-lactamase inhibitors, for algorithm-based detection and classification of CPOs. That is, every Gram-negative isolate is tested for carbapenemase production.

The Rapidec Carba NP test (bioMérieux, St. Louis, MO) is a representative of the current state of the art for rapid manual carbapenemase confirmatory tests. It is typically performed following the detection of carbapenem nonsusceptibility after the completion of the routine susceptibility test. The current study was designed to evaluate the performances of BD Phoenix CPO Detect and Rapidec Carba NP against a previously characterized challenge set of isolates that included organisms of high diagnostic difficulty for many currently used carbapenemase tests.

## RESULTS

### Carbapenemase detection.

Both tests yielded positive results for at least 97% of the CPOs (Table 1). The overall sensitivities for detection of all CPOs were BD Phoenix CPO Detect, 97.1% (95% confidence interval [95% CI], 94.1% to 98.6%); Rapidec Carba NP tests with interpretation 1 (borderline results interpreted as positive), 98.8% (95% CI, 96.4% to 99.6%); and Rapidec Carba NP tests with interpretation 2 (borderline results interpreted as negative), 97.1% (95% CI, 94.1% to 98.6%). Of the 110 class-A-producing CPOs, 107 (97.3%) were positive in BD Phoenix CPO Detect tests and 100% (110/110) and 98.2% (108/110) were positive in Rapidec Carba NP tests with interpretations 1 and 2, respectively. For class-B-producing CPOs, BD Phoenix CPO Detect was positive for 87/91 (95.6%) of isolates and Rapidec Carba NP was positive for 90/91 (98.9%) with both interpretations (i.e., no borderline results), and for class-D-producing CPOs, BD Phoenix CPO Detect was positive for 35/35 (100%) of isolates and Rapidec Carba NP was positive for 33/35 (94.3%) (no borderline results). The seven dual-carbapenemase producers were all positive by both tests. Notable examples of carbapenemase detection of highly challenging isolates by both tests included the detection of KPC production by Acinetobacter baumannii and P. aeruginosa isolates and the high detection rates for OXA (class D) carbapenemases. Other notable results were detection of Klebsiella pneumoniae KPC-4 production by BD Phoenix CPO Detect and of Proteus mirabilis IMP-27 production by Rapidec Carba NP.

**TABLE 1:** Sensitivity and specificity of CPO detection (the positive/negative testing phase of the study)

Evaluation criterion	Carbapenemase producer type or status	No. tested	% correct results (95% confidence interval)		
			BD Phoenix CPO Detect	Rapidec Carba NP interpretation^*a*^	
				1	2
Sensitivity	Class A	110	97.3	100	98.2
	Class B	91	95.6	98.9	98.9
	Class D	35	100	94.3	94.3
	Dual carbapenemase	7	All positive^*b*^	All positive^*b*^	All positive^*b*^
	All CPOs	243	97.1 (94.1–98.6)	98.8 (96.4–99.6)	97.1 (94.1–98.6)
Specificity	All non-CPOs	51	68.6 (54.9–79.7)	60.8 (47.1–73.0)	78.4 (65.4–87.5)

a Interpretation 1, borderline results interpreted as positive; interpretation 2, borderline results interpreted as negative.

b All 7 dual carbapenemase producers yielded a positive result.

Falsely negative results occurred in both tests with one of two KPC-4-producing K. pneumoniae isolates. BD Phoenix CPO Detect also yielded falsely negative results with two KPC producers (a carbapenem-susceptible Citrobacter freundii isolate and an ertapenem-resistant, imipenem- and meropenem-susceptible Klebsiella oxytoca isolate) and with four class-B CPOs (an IMP-8-producing Enterobacter cloacae isolate, two VIM-producing P. aeruginosa isolates and an IMP-27-producing P. mirabilis isolate). Rapidec Carba NP yielded falsely negative results with a second isolate of KPC-4-producing K. pneumoniae, an OXA-48-producing K. pneumoniae isolate and an OXA-181-producing K. pneumoniae isolate. It also produced an uninterpretable result with an NDM-producing K. pneumoniae isolate.

In tests with non-CPOs, BD Phoenix CPO Detect accurately yielded negative results for 35/51 of isolates; i.e., 68.6% specificity (95% CI, 54.9% to 79.7%). In Rapidec Carba NP tests there was a 17.6% difference in accuracy between the two interpretations. With interpretation 1, 31/51 of results were correctly interpreted as negative, i.e., 60.8% specificity (95% CI, 47.1% to 73.0%), whereas with interpretation 2, where borderline results were interpreted as negative, more results (40/51) were correctly negative; i.e., 78.4% specificity (95% CI, 65.4% to 87.5%). AmpC production was associated with falsely positive results for 11 isolates in the BD Phoenix CPO Detect tests and for 12 isolates in the Rapidec Carba NP tests. One SHV-18-producing K. pneumoniae isolate yielded an uninterpretable result with the Rapidec Carba NP test. Causes of falsely positive results other than association with AmpC production were not investigated.

### Carbapenemase classifications.

The BD Phoenix CPO Detect correctly classified 91 of the 107 class A producers (85.0%) that yielded a positive carbapenemase test (Table 2). It correctly classified 63 of the detected 87 class B producers (72.4%) and 31 of the 35 class D producers (88.6%). Positive unclassified results were obtained with an additional 13 Class A carbapenemase producers, 22 Class B producers and 2 Class D producers. In tests with dual-carbapenemase-producing isolates, only one carbapenemase classification interpretation per organism was provided (see Table 2 footnote). Two isolates had a positive untyped interpretation, four isolates were given a class D CPO classification and one was given a class B CPO classification. The class D and B interpretations correctly indicated one of the two carbapenemases in these isolates. No class B carbapenemase producers were misclassified as class A producers.

**TABLE 2:** Results of carbapenemase classifications by BD Phoenix CPO Detect

CPO type	No. tested	No. of positive carbapenemase tests	Classification by BD Phoenix CPO Detect			
			Class A	Class B	Class D	Unclassified positive
Class A	110	107	91	3	0	13
Class B	91	87	0	63	2	22
Class D	35	35	1	1	31	2
Dual^*a*^	7	7	0	1	4	2

a The classification results for the dual carbapenemase producers were as follows: positive unclassified, A. baumannii (OXA-23 + OXA-40) and E. cloacae (KPC-18 + VIM-1); class D, A. baumannii (OXA-23 + NDM), two isolates of K. pneumoniae (OXA-181 + NDM), and K. pneumoniae (OXA-232 + NDM); and class B, E. cloacae (KPC-18 + VIM-1).

### Impact on Workflow.

The BD Phoenix CPO Detect required less hands-on time than the Rapidec Carba NP in part because it did not require additional manual operations after loading an inoculated panel into the instrument. BD Phoenix CPO Detect hands-on time per test was 1 min 34 s. Two hands-on times per test were determined for the Rapidec Carba NP. If the test was positive after the initial 30-min incubation period (i.e., completed), the hands-on time per test was 2 min 3 s. If the test was negative at this time, additional handling and incubation was required, which extended the hands-on time per test to 2 min 24 s. An additional component for the Rapidec Carba NP that was not included in this study is the hands-on time to set up a susceptibility test (if needed) prior to doing the Rapidec Carba NP test. This is not necessary for BD Phoenix CPO Detect, as the carbapenemase test is included in the susceptibility test.

## DISCUSSION

The high mortality and continuing emergence of resistance associated with CPO infections make it imperative for laboratories to have the capability to provide rapid, accurate CPO detection (12). This capability, in conjunction with good infection control, can help to keep medical institutions at minimal risk from the CPO pandemic.

Previous evaluations of the Rapidec Carba NP test have reported both high sensitivity and specificity (15–18). To date there are no published evaluations of the BD Phoenix CPO Detect test. A 2017 conference presentation reported a multicenter evaluation of over 1,000 fresh clinical and frozen isolates in which the BD Phoenix CPO Detect had similar performance characteristics to the previously reported Rapidec Carba NP results, exceeding 99% sensitivity and 94% specificity (S. Chandrasekaran, H. K. Huse, G. A. Denys, X. Li, S. Miller, J. Hindler, and R. M. Humphries, presented at ASM Microbe, New Orleans, LA, 2017). In the current study, both tests exhibited high overall carbapenemase detection capabilities (>97% for all types of CPOs). This was comparable to previous reports for the Rapidec Carba NP, but the lower specificity in this study reflected the inclusion of isolates chosen to provide maximum diagnostic difficulty. The high overall sensitivity of both tests reflects good detection capabilities with isolates of both Enterobacteriaceae and the nonfermenters P. aeruginosa and A. baumannii. Detection of the carbapenemases of nonfermenters can be a technically difficult challenge (19, 20). A meaningful comparison of how the tests performed against Enterobacteriaceae versus nonfermenters is not possible for this study, as the two organism groups were not comparable in their numbers of isolates and types of β-lactamase production (see supplemental material).

The differences between the specificity values of the current study and other studies demonstrates the impact of isolate selection on the findings of evaluation studies. The testing of mostly fresh clinical isolates supplemented with isolates possessing relevant resistance mechanisms can be helpful for determining the suitability of a test for clinical laboratories. More stringent evaluations, such as the current study, that include a high proportion of difficult isolates are more likely to discover the weaknesses and limitations of tests. Both types of studies can be valuable. Ideally, the more stringent type of evaluation should be performed as early as possible in test development to alert users and manufacturers to the strengths and weaknesses of the test.

The greatest value of a carbapenemase classification test lies in its application to empirical antibiotic therapy. The detection of a class A CPO means that ceftazidime/avibactam is a potential candidate for therapy and therefore should not be discounted as an option. In contrast, detection of a class B CPO contraindicates therapy with ceftazidime/avibactam. The detection of a class A CPO is not an unequivocal indication to treat with ceftazidime/avibactam. Nor is it a substitute for antibiotic susceptibility testing. In this study, no class B CPO was misclassified as a class A CPO. If it had occurred, it would constitute a potentially harmful error as it could mislead a clinician to empirically prescribe ceftazidime/avibactam for an infection in which the pathogen would be resistant. As backup, the BD Phoenix CPO Detect is currently accompanied by a ceftazidime/avibactam susceptibility test and should accommodate additional newer β-lactamase inhibitor combinations as they become available.

In conclusion, the current study confirmed that both tests have the speed and high sensitivity that is needed to rapidly detect carbapenemases, and the BD Phoenix CPO Detect is additionally capable of classifying the carbapenemases of many CPOs. Both tests exhibited specificity issues that are likely to be encountered only infrequently in most clinical laboratories. The Rapidec Carba NP has an interpretation issue with isolates that yield borderline results. Such isolates are probably only rarely encountered and a possible strategy would be to report borderline results as indeterminate and to use an alternative method for retesting. A potential advantage of the Rapidec Carba NP is its speed of CPO test results when used without performing an initial susceptibility test (only 10 to 30 min for some isolates). This could be advantageous in circumstances of high urgency, when testing isolates from previously positive patients, or in surveillance testing.

Overall, both tests offer major advances in their combination of speed, convenience and sensitivity compared to tests that require overnight incubation. The BD Phoenix CPO Detect is a unique test in that it is the first automated carbapenemase test and is included in the routine Phoenix susceptibility test to save time and avoid reliance on individuals to decide if a carbapenemase detection test is warranted. In addition, it has the ability to classify carbapenemases, which can be clinically important for identifying possible therapeutic choices.

## MATERIALS AND METHODS

### Isolates.

The 294 study isolates consisted of Enterobacteriaceae (*n* = 241), P. aeruginosa (*n* = 29), and A. baumannii (*n* = 24) isolates that were previously characterized by PCR, microarray, DNA sequencing, whole-genome sequencing, and phenotypic and biochemical tests for types of β-lactamase production. Isolates were chosen to provide extreme diagnostic difficulty, e.g., producers of OXA carbapenemases (19, 21), KPC-producing A. baumannii (20), high-level AmpC- and metallo-β-lactamase-producing isolates that yield inaccurate results with the modified Hodge test (22–24), carbapenem-susceptible CPOs (5, 25), mucoid CPOs (15), and carbapenemase-negative isolates that produce an ESBL and have a porin mutation (26–28). They were not routine clinical isolates. They included 110 isolates producing class A carbapenemases, including KPC, NMC-A, IMI, and SME enzymes; 91 isolates producing class B carbapenemases, including NDM, SPM, IMP, and VIM enzymes; 35 isolates producing class D carbapenemases, including OXA-23, 40, 48, 58, 72, 181, and 232; and seven isolates producing two carbapenemases. Also tested were 51 carbapenemase-negative isolates that produced ESBLs, AmpCs (including hyperproducers), K1, broad-spectrum β-lactamases, and porin mutants. The isolates were provided from culture collections at the University of Louisville and the Centers for Disease Control and Prevention and Food and Drug Administration Antimicrobial Resistance Isolate Bank. Table S1 in the supplemental material contains a list of the individual isolates, resistance mechanisms, and the study results for each isolate.

### BD Phoenix CPO Detect and Rapidec Carba NP tests.

**FIG 1:**
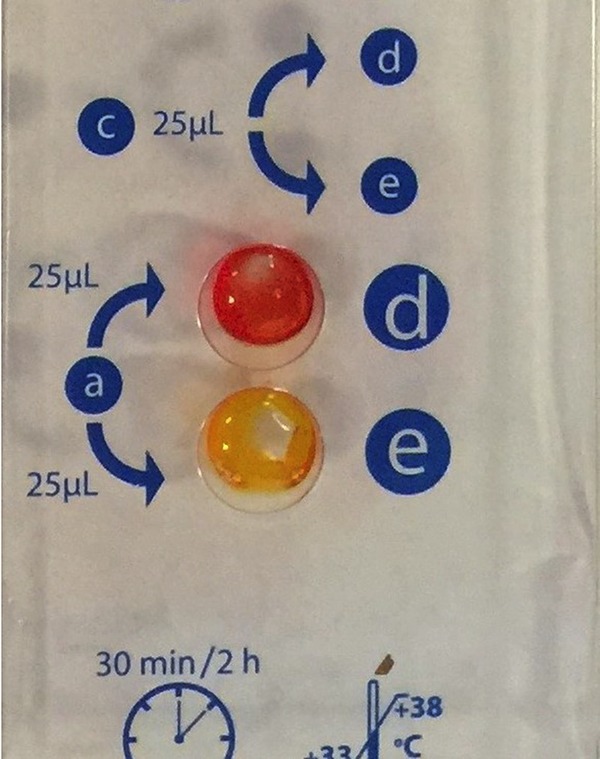
Representative photo of positive Rapidec Carba NP test result exhibiting a significant color variation between wells d (test control) and e (test isolate). The isolate is KPC-producing K. pneumoniae BAA-1705.

Inocula were prepared from overnight growth on blood agar plates (BD Diagnostics Systems, Sparks, MD) and harvested from growth adjacent to imipenem disks (BD Diagnostics Systems, Sparks, MD). Both tests were performed blinded and according to the manufacturers' recommendations. BD Phoenix CPO Detect results were interpreted by a BD Phoenix algorithm and Rapidec Carba NP results were interpreted according to the manufacturer's definitions that a positive test exhibits a “significant variation in color” between the test and test control wells (Fig. 1) and an uninterpretable result is one in which the control well is any color other than red or orange, or if the control well is orange and the test well is red. The BD Phoenix CPO Detect panel also provided MICs of ertapenem, imipenem, and meropenem. These are included in the supplemental material.

**FIG 2:**
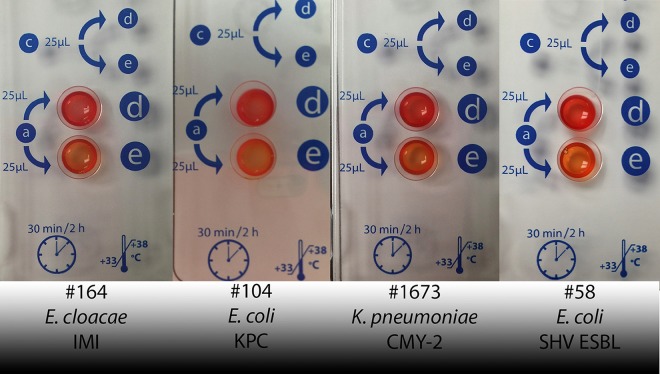
Representative photos of difficult-to-interpret Rapidec Carba NP test results. Well d is the test control and e is the test well. Isolates 164 and 104 are E. cloacae and E. coli that produce IMI and KPC class A carbapenemases, respectively, for which the correct test interpretation is positive, which means that they exhibit significant color variations. Isolates 1673 and 58 are K. pneumoniae and E. coli that produce CMY-2 and an ESBL, respectively, for which the correct interpretation is negative, which means that they do not exhibit significant color variations. The ill-defined boundary between positive and negative interpretations for these and other similar isolates necessitated the interpretation of borderline results as both positive and negative to provide two sets of Rapidec Carba NP results. Interpretation 1 was a positive interpretation for borderline results and interpretation 2 was a negative interpretation.

Because it was difficult to distinguish between significant and insignificant variations in color for borderline Rapidec Carba NP results, two sets of interpretations were used to produce two sets of results. Using interpretation 1, borderline results were interpreted as positive and using interpretation 2, borderline results were interpreted as negative. Figure 2 shows four representative borderline results to illustrate the difficulty of interpretation.

### Evaluation criteria.

Both tests were evaluated for accuracy of carbapenemase detection and timed to assess workflow impact (i.e., requirement for hands-on time). BD Phoenix CPO Detect was also evaluated for ability to classify the carbapenemases of positive isolates.

## Supplementary Material

Supplemental material
