# Supplementary material for: High-Stringency Evaluation of the Automated BD Phoenix CPO Detect and Rapidec Carba NP Tests for Detection and Classification of Carbapenemases
Source: J Clin Microbiol. 2017 Nov 27;55(12):3437–43. doi: 10.1128/JCM.01215-17 (PMC5703810; doi:10.1128/JCM.01215-17)
Supplement: Supplemental material [file supp_55_12_3437__index.html]

Supplemental material 

# High-Stringency Evaluation of the Automated BD Phoenix CPO Detect and Rapidec Carba NP Tests for Detection and Classification of Carbapenemases

## Supplemental material

- Supplemental file 1 -

  Table S1 (Isolate information and results for Rapidec Carba NP and BD Phoenix CPO Detect panels, with carbapenem MICs for ertapenem, imipenem, and meropenem)

  PDF, 1.2M
