# Supplementary material for: High-Stringency Evaluation of the Automated BD Phoenix CPO Detect and Rapidec Carba NP Tests for Detection and Classification of Carbapenemases
Source: J Clin Microbiol. 2017 Nov 27;55(12):3437–43. doi: 10.1128/JCM.01215-17 (PMC5703810; doi:10.1128/JCM.01215-17)
Supplement: Supplemental material [file JCM.01215-17_zjm012175720s1.pdf]

Table S1. Isolate Information and Results for Rapidec Carba NP (30 minute & 2 hour incubations) and BD Phoenix CPO Detect (Phx) Panels with Carbapenem MICs in µg/ml for Ertapenem (ETP), Imipenem (IPM) & meropenem (MEM)

Organism	Mechanism(s)	Rapidec 30 mins	Rapidec 2h	Phx Pos/Neg Result	Phx Classification	Phx ETP MIC	Phx IPM MIC	Phx MEM MIC
Citrobacter amalonaticus	KPC-2-like	+		pos	A	> 1	> 8	> 8
C. freundii	KPC	+		neg	neg	1	2	0.25
C. freundii	KPC	+		pos	A	> 1	4	8
C. freundii	KPC-2	+		pos	A	> 1	> 8	4
C. freundii	NDM-1	+		pos	B	> 1	> 8	> 8
C. freundii	NDM-1	+		pos	B	> 1	> 8	> 8
C. freundii	NDM-1,CTX-M-15,TEM-1,OXA-1	+		pos	B	> 1	> 8	> 8
C. freundii complex	KPC-3 like	+		pos	A	> 1	8	8
Citrobacter sp.	NDM	+		pos	B	> 1	> 8	> 8
C. freundii	Derepressed AmpC	-	-	neg	neg	<=0.25	1	<=0.125
Enterobacter aerogenes	IMP	+		pos	B	> 1	> 8	8
E. aerogenes	OXA-48	-	+	pos	D	> 1	4	2
E. aerogenes	cAmpC	-	-	neg	neg	> 1	1	0.5
E. aerogenes	Hi AmpC	-	+	pos	B	> 1	> 8	> 8
E. aerogenes	Hi AmpC,SHV-5-like	-	+	neg	neg	<=0.25	1	<=0.125
E. cloacae	KPC-18,VIM-1	+		pos	pos - untyped	> 1	> 8	8
E. cloacae	KPC-18,VIM-1	+		pos	B	> 1	> 8	> 8
E. cloacae	IMP-8	+		neg	neg	0.5	4	0.5
E. cloacae	NDM	+		pos	pos - untyped	> 1	> 8	<=0.125
E. cloacae	NDM	+		pos	B	> 1	> 8	> 8
E. cloacae	NDM	+		pos	B	> 1	> 8	> 8
E. cloacae	VIM	+		pos	B	> 1	8	> 8
E. cloacae	IMI	+		pos	A	> 1	> 8	> 8
E. cloacae complex	IMI	+		pos	A	> 1	> 8	> 8
E. cloacae	KPC	+		pos	A	> 1	> 8	> 8
E. cloacae	KPC	+		pos	A	> 1	4	2
E. cloacae	KPC	-	+	pos	A	> 1	> 8	8

Organism	Mechanism(s)	Rapidec 30 mins	Rapidec 2h	Phx Pos/Neg Result	Phx Classification	Phx ETP MIC	Phx IPM MIC	Phx MEM MIC
E. cloacae	KPC	-	(w)*	pos	A	1	4	0.5
E. cloacae	KPC	+		pos	A	> 1	> 8	> 8
E. cloacae	KPC; CTX-M-1; ACT/MIR	+		pos	A	> 1	8	4
E. cloacae	KPC	+		pos	A	> 1	8	8
E. cloacae	KPC	+		pos	A	> 1	4	> 8
E. cloacae	KPC	+		pos	A	> 1	8	8
E. cloacae	KPC	-	+	pos	A	> 1	8	4
E. cloacae	KPC	-	+	pos	A	> 1	8	8
E. cloacae	KPC	+		pos	A	> 1	> 8	> 8
E. cloacae	KPC	+		pos	A	> 1	> 8	> 8
E. cloacae	KPC-2	+		pos	A	> 1	> 8	> 8
E. cloacae	KPC-2-like	+		pos	A	> 1	2	2
E. cloacae	KPC-2-like	+		pos	A	> 1	> 8	> 8
E. cloacae	KPC-2-like	+		pos	A	> 1	1	1
E. cloacae	KPC-2-like	+		pos	A	> 1	8	4
E. cloacae	KPC-3 TEM-1	+		pos	A	> 1	8	8
E. cloacae	KPC 3 TEM-1	+		pos	B	> 1	4	2
E. cloacae	NMC-A	+		pos	A	> 1	> 8	> 8
E. cloacae	cAmpC	+*		neg	neg	> 1	1	0.5
E. cloacae	cAmpC	-	-	neg	neg	> 1	NONE	2
E. cloacae	cAmpC	-	-	neg	neg	1	1	0.25
E. cloacae	cAmpC	-	(w)	neg	neg	<=0.25	<=0.25	<=0.125
E. cloacae	Hi AmpC	-	-	pos	B	> 1	> 8	> 8
E. cloacae	Hi AmpC	-	-	neg	neg	> 1	> 8	8
E. cloacae	Hi AmpC	-	+	pos	B	> 1	> 8	> 8
Escherichia coli	NDM	+		pos	B	> 1	> 8	> 8
E. coli	NDM	+		pos	B	> 1	> 8	> 8
E. coli	NDM	+		pos	B	> 1	> 8	> 8
E. coli	NDM	+		pos	B	> 1	> 8	> 8
E. coli	NDM	+		pos	B	> 1	> 8	> 8

Organism	Mechanism(s)	Rapidec 30 mins	Rapidec 2h	Phx Pos/Neg Result	Phx Classification	Phx ETP MIC	Phx IPM MIC	Phx MEM MIC
E. coli	NDM	+		pos	D	> 1	> 8	> 8
E. coli	NDM-1	+		pos	B	> 1	> 8	> 8
E. coli	NDM-1,CTX-M-15	+		pos	B	> 1	> 8	> 8
E. coli	NDM-1,CTX-M-15, TEM-1	+		pos	B	> 1	> 8	> 8
E. coli	NDM, CTX-M-1	+		pos	B	> 1	> 8	> 8
E. coli	NDM	+		pos	B	> 1	> 8	> 8
E. coli	NDM-1	+		pos	B	> 1	> 8	> 8
E. coli	NDM-5	+		pos	B	> 1	> 8	> 8
E. coli	NDM-5	+		pos	B	> 1	> 8	> 8
E. coli	NDM-6	+		pos	D	> 1	> 8	> 8
E. coli	KPC	+		pos	A	> 1	4	8
E. coli	KPC	-	(w)	pos	A	> 1	> 8	2
E. coli	KPC-3	+		pos	A	> 1	4	2
E. coli	KPC-3 TEM-1	+		pos	A	> 1	4	8
E. coli	KPC-3 like	+		pos	A	> 1	8	8
E. coli	KPC-3 like	+		pos	A	> 1	8	4
E. coli	CMY-2 type	-	-	neg	neg	<=0.25	> 8	> 8
E. coli	CMY-2 type	-	-	neg	neg	<=0.25	0.5	<=0.125
E. coli	Hi AmpC	-	+	neg	neg	<=0.25	<=0.25	<=0.125
E. coli	Hi AmpC,TEM-1-like	-	+	neg	neg	> 1	> 8	4
E. coli	Hi AmpC, SHV-12 like	-	+	pos	B	> 1	> 8	> 8
E. coli	DHA-like	-	-	neg	neg	> 1	> 8	2
E. coli	cAmpC	-	+	neg	neg	<=0.25	<=0.25	<=0.125
E. coli	Hi AmpC	-	+	pos	B	> 1	> 8	> 8
E. coli	LAT-4	-	+	neg	neg	0.5	1	<=0.125
E. coli	Hi AmpC,TEM-1-like	-	+	neg	neg	> 1	> 8	2
E. coli	CMY-2	-	-	neg	neg	<=0.25	0.5	<=0.125
E. coli	CMY-2	-	-	neg	neg	> 1	2	2
E. coli	CTX-M-12	-	+	neg	neg	<=0.25	<=0.25	<=0.125

Organism	Mechanism(s)	Rapidec 30 mins	Rapidec 2h	Phx Pos/Neg Result	Phx Classification	Phx ETP MIC	Phx IPM MIC	Phx MEM MIC
E. coli	164S (TEM)	-	+	neg	neg	<=0.25	0.5	<=0.125
E. coli	CTX-M9	-	-	pos	B	> 1	> 8	> 8
E. coli	ESBL	-	+	pos	D	> 1	<=0.25	0.5
E. coli	High TEM-1	-	-	neg	neg	<=0.25	<=0.25	<=0.125
Hafnia alvei	KPC	+		pos	A	> 1	8	2
Klebsiella oxytoca	KPC	+		neg	neg	> 1	> 8	> 8
K. oxytoca	KPC	+		pos	A	> 1	8	8
K. oxytoca	KPC	+		pos	A	> 1	8	8
K. oxytoca	KPC	-	+	pos	pos - untyped	> 1	> 8	> 8
K. oxytoca	DHA-like, SHV ESBL, TEM-1-like, K1	-	-	neg	neg	<=0.25	0.5	<=0.125
K. oxytoca	K1 hyperproducer	-	-	neg	neg	<=0.25	0.5	<=0.125
K. ozaenae	KPC	+		pos	B	> 1	> 8	> 8
K. ozaenae	OXA-181	-	(w)	pos	D	> 1	8	8
K. pneumoniae	IMP	-	+	pos	B	> 1	> 8	> 8
K. pneumoniae	IMP	+		pos	pos - untyped	> 1	8	> 8
K. pneumoniae	IMP-8	+		pos	B	> 1	8	4
K. pneumoniae	NDM-1	+		pos	pos - untyped	> 1	> 8	> 8
K. pneumoniae	NDM	+		pos	B	> 1	> 8	> 8
K. pneumoniae	NDM	+		pos	B	> 1	> 8	> 8
K. pneumoniae	NDM	+		pos	B	> 1	> 8	> 8
K. pneumoniae	NDM, OXA-181	+		pos	D	> 1	> 8	8
K. pneumoniae	NDM, OXA-232	+		pos	D	> 1	> 8	> 8
K. pneumoniae	NDM	+		pos	B	> 1	> 8	> 8
K. pneumoniae	NDM	+		pos	B	> 1	> 8	> 8
K. pneumoniae	NDM	+		pos	B	> 1	> 8	> 8
K. pneumoniae	NDM	+		pos	pos - untyped	> 1	> 8	> 8
K. pneumoniae	NDM	+		pos	pos - untyped	> 1	> 8	> 8
K. pneumoniae	NDM	+		pos	pos - untyped	> 1	> 8	> 8
K. pneumoniae	NDM-1	+		pos	B	> 1	> 8	> 8

Organism	Mechanism(s)	Rapidec 30 mins	Rapidec 2h	Phx Pos/Neg Result	Phx Classification	Phx ETP MIC	Phx IPM MIC	Phx MEM MIC
K. pneumoniae	NDM	U	U*	pos	B	>1	>8	>8
K. pneumoniae	NDM-1	+		pos	B	> 1	> 8	> 8
K. pneumoniae	NDM-1	+		pos	pos - untyped	> 1	> 8	> 8
K. pneumoniae	NDM-1,CTX-M-15-like,SHV-1-like	+		pos	B	> 1	> 8	> 8
K. pneumoniae	NDM-1,CTX-M-15-like,SHV-1-like	+		pos	B	> 1	8	8
K. pneumoniae	NDM-1, CTX-M-15,OXA-181	+		pos	D	> 1	> 8	> 8
K. pneumoniae	NDM-1,CTX-M-15,TEM-1,OXA-1	+		pos	B	> 1	> 8	> 8
K. pneumoniae	NDM-1,CTX-M-15,CMY-16,TEM-1	+		pos	pos - untyped	> 1	> 8	> 8
K. pneumoniae	VIM	+		pos	pos - untyped	> 1	> 8	> 8
K. pneumoniae	VIM	+		pos	pos - untyped	> 1	> 8	> 8
K. pneumoniae	VIM	+		pos	B	> 1	> 8	> 8
K. pneumoniae	VIM	+		pos	B	> 1	> 8	> 8
K. pneumoniae	VIM-like,SHV-12-like,SHV-1-like	+		pos	B	> 1	> 8	> 8
K. pneumoniae	VIM-1	+		pos	B	> 1	> 8	> 8
K. pneumoniae	KPC	+		pos	pos - untyped	> 1	> 8	> 8
K. pneumoniae	KPC	+		pos	A	> 1	8	8
K. pneumoniae	KPC	+		pos	A	> 1	> 8	> 8
K. pneumoniae	KPC	+		pos	A	> 1	> 8	> 8
K. pneumoniae	KPC	-	+	pos	A	> 1	> 8	> 8
K. pneumoniae	KPC	+		pos	A	> 1	> 8	> 8
K. pneumoniae	KPC	+		pos	A	> 1	> 8	> 8
K. pneumoniae	KPC	+		pos	A	> 1	> 8	> 8
K. pneumoniae	KPC	+		pos	A	> 1	> 8	> 8
K. pneumoniae	KPC	+		pos	A	> 1	> 8	> 8

Organism	Mechanism(s)	Rapidec 30 mins	Rapidec 2h	Phx Pos/Neg Result	Phx Classification	Phx ETP MIC	Phx IPM MIC	Phx MEM MIC
K. pneumoniae	KPC	+		pos	pos - untyped	> 1	> 8	> 8
K. pneumoniae	KPC	+		pos	pos - untyped	> 1	> 8	> 8
K. pneumoniae	KPC	+		pos	A	> 1	4	NONE
K. pneumoniae	KPC	+		pos	A	> 1	> 8	> 8
K. pneumoniae	KPC	+		pos	A	> 1	> 8	8
K. pneumoniae	KPC	+		pos	A	> 1	4	2
K. pneumoniae	KPC	-	+	pos	A	> 1	> 8	> 8
K. pneumoniae	KPC	+		pos	A	> 1	8	> 8
K. pneumoniae	KPC	+		pos	A	> 1	> 8	> 8
K. pneumoniae	KPC	+		pos	A	> 1	> 8	> 8
K. pneumoniae	KPC	+		pos	A	> 1	8	> 8
K. pneumoniae	KPC-2-like	+		pos	A	> 1	> 8	> 8
K. pneumoniae	KPC-2-like	+		pos	A	> 1	> 8	> 8
K. pneumoniae	KPC-2-like	+		pos	A	> 1	> 8	> 8
K. pneumoniae	KPC-2-like	+		pos	A	> 1	> 8	> 8
K. pneumoniae	KPC-2-like	+		pos	A	> 1	8	8
K. pneumoniae	KPC-2-like	+		pos	A	> 1	> 8	> 8
K. pneumoniae	KPC-2-like	+		pos	A	> 1	8	4
K. pneumoniae	KPC-2-like	+		pos	A	> 1	> 8	> 8
K. pneumoniae	KPC-2-like	+		pos	A	> 1	> 8	> 8
K. pneumoniae	KPC-2-like	+		pos	A	> 1	8	4
K. pneumoniae	KPC-2	+		pos	A	> 1	> 8	> 8
K. pneumoniae	KPC-3	+		pos	A	> 1	> 8	> 8
K. pneumoniae	KPC-3	+		pos	pos - untyped	> 1	> 8	> 8
K. pneumoniae	KPC-3	+		pos	A	> 1	8	> 8
K. pneumoniae	KPC-3	+		pos	A	> 1	> 8	> 8
K. pneumoniae	KPC-3	+		pos	A	> 1	> 8	> 8
K. pneumoniae	KPC-2	+		pos	A	> 1	> 8	> 8
K. pneumoniae	KPC-3	+		pos	A	> 1	> 8	> 8
K. pneumoniae	KPC-3	+		pos	A	> 1	> 8	> 8

Organism	Mechanism(s)	Rapidec 30 mins	Rapidec 2h	Phx Pos/Neg Result	Phx Classification	Phx ETP MIC	Phx IPM MIC	Phx MEM MIC
K. pneumoniae	KPC-2, SHV-5-like,TEM-1-like	+		pos	pos - untyped	> 1	> 8	> 8
K. pneumoniae	KPC-3 like	+		pos	A	> 1	8	> 8
K. pneumoniae	KPC-3,SHV-12-like,SHV-1, OXA-9-like,TEM-1-like	+		pos	A	> 1	4	4
K. pneumoniae	KPC-4	-	+	pos	A	> 1	1	0.5
K. pneumoniae	KPC-4	-	+	neg	neg	> 1	4	2
K. pneumoniae	KPC-6	+		pos	A	> 1	4	4
K. pneumoniae	KPC-8	+		pos	pos - untyped	> 1	> 8	> 8
K. pneumoniae	KPC-8	+		pos	pos - untyped	> 1	> 8	> 8
K. pneumoniae	OXA-48	+		pos	D	> 1	4	2
K. pneumoniae	OXA-48	-	+	pos	D	> 1	> 8	8
K. pneumoniae	OXA-48	-	+	pos	D	> 1	4	2
K. pneumoniae	OXA-48	-	+	pos	D	> 1	4	1
K. pneumoniae	OXA-48	-	+	pos	D	> 1	> 8	> 8
K. pneumoniae	OXA-48	-	+	pos	D	> 1	> 8	> 8
K. pneumoniae	OXA-48	-	+	pos	D	> 1	> 8	> 8
K. pneumoniae	OXA-48	-	+	pos	D	> 1	4	2
K. pneumoniae	OXA-48	-	+	pos	D	> 1	2	2
K. pneumoniae	OXA-48	-	-	pos	D	> 1	4	4
K. pneumoniae	OXA-48	-	+	pos	D	> 1	4	8
K. pneumoniae	OXA-48	-	+	pos	D	> 1	> 8	> 8
K. pneumoniae	OXA-181	-	+	pos	D	> 1	8	4
K. pneumoniae	OXA-181	-	+	pos	D	> 1	4	4
K. pneumoniae	OXA-181	+		pos	D	> 1	4	2
K. pneumoniae	OXA-181	-	-	pos	D	> 1	4	4
K. pneumoniae	OXA-232	-	+	pos	D	> 1	8	> 8
K. pneumoniae	OXA-232	-	+	pos	D	> 1	> 8	> 8
K. pneumoniae	OXA-181	+		pos	D	> 1	> 8	> 8
K. pneumoniae	LAT-4	-	-	neg	neg	0.5	0.5	<=0.125

Organism	Mechanism(s)	Rapidec 30 mins	Rapidec 2h	Phx Pos/Neg Result	Phx Classification	Phx ETP MIC	Phx IPM MIC	Phx MEM MIC
K. pneumoniae	FOX-5, SHV-4 like	-	-	neg	neg	<=0.25	<=0.25	<=0.125
K. pneumoniae	TEM-1,SHV-1, OXA-9, SHV-5 ACT-1	-	-	neg	neg	0.5	2	<=0.125
K. pneumoniae	CMY-2-like	-	-	pos	pos - untyped	> 1	> 8	> 8
K. pneumoniae	CMY-2, SHV-1, TEM-1-like	-	-	neg	neg	<=0.25	<=0.25	<=0.125
K. pneumoniae	MOX-1	-	-	neg	neg	> 1	<=0.25	0.25
K. pneumoniae	FOX-1	-	-	neg	neg	<=0.25	<=0.25	<=0.125
K. pneumoniae	SHV-18	U	U	neg	neg	<=0.25	<=0.25	<=0.125
K. pneumoniae	CTX-M28, OMPK36, OMPK-35	+		pos	pos - untyped	> 1	1	4
K. pneumoniae	SHV-12,OMPK-36	-	-	pos	B	> 1	> 8	> 8
K. pneumoniae	OMP-K35	-	+	pos	pos - untyped	> 1	> 8	> 8
K. pneumoniae	CTX-M14; DHA-1, OMPK35	-	-	pos	pos - untyped	> 1	> 8	> 8
K. pneumoniae	SHV	-	-	neg	neg	<=0.25	<=0.25	<=0.125
K. pneumoniae	CTX-M2, OMPK-36	-	-	pos	pos - untyped	> 1	8	> 8
K. pneumoniae	TEM-1, SHV-1, CTX-M-15	-	-	pos	pos - untyped	> 1	4	8
K. pneumoniae	TEM-1, SHV-1, CTX-M-15	-	+	pos	pos - untyped	> 1	> 8	> 8
Kluyvera ascorbata	KPC	+		pos	pos - untyped	> 1	> 8	> 8
Morganella morganii	NDM	+		pos	pos - untyped	> 1	> 8	4
M. morganii	KPC	(w)	+	pos	A	> 1	> 8	4
M. morganii	Inducible AmpC	-	+	neg	neg	<=0.25	4	<=0.125
M. morganii	Derepressed AmpC	+		pos	pos - untyped	> 1	4	4
Proteus mirabilis	IMP-27	-	+	neg	neg	> 1	8	> 8
P. mirabilis	NDM	-	+	pos	B	> 1	> 8	> 8
P. mirabilis	KPC	-	+	pos	pos - untyped	> 1	> 8	8
P. mirabilis	KPC	-	+	pos	B	0.5	8	0.25

Organism	Mechanism(s)	Rapidec 30 mins	Rapidec 2h	Phx Pos/Neg Result	Phx Classification	Phx ETP MIC	Phx IPM MIC	Phx MEM MIC
P. mirabilis	CMY-like, TEM-1-like	-	-	pos	B	<=0.25	8	<=0.125
Providencia rettgeri	NDM	-	+	pos	B	> 1	> 8	> 8
P. rettgeri	NDM-1	-	+	pos	B	> 1	> 8	> 8
Raoultella ornithinolytica	KPC	+		pos	A	> 1	8	4
Salmonella senftenberg	NDM	+		pos	B	> 1	> 8	> 8
Serratia marcescens	IMP-1	+		pos	pos - untyped	> 1	> 8	> 8
S. marcescens	NDM-1	+		pos	B	> 1	> 8	> 8
S. marcescens	KPC	+		pos	pos - untyped	> 1	> 8	> 8
S. marcescens	SME	+		pos	A	> 1	> 8	> 8
S. marcescens	SME	(w)	+	pos	A	> 1	> 8	> 8
S. marcescens	SME	+		pos	A	> 1	> 8	> 8
S. marcescens	SME	+		pos	A	> 1	> 8	> 8
S. marcescens	SME	+		pos	A	> 1	> 8	> 8
S. marcescens	SME	+		pos	A	> 1	> 8	> 8
S. marcescens	SME-like	+		pos	A	> 1	> 8	> 8
S. marcescens	SME-like	+		pos	A	> 1	> 8	> 8
S. marcescens	SME	+		pos	A	> 1	> 8	> 8
S. marcescens	SME	+		pos	A	> 1	> 8	8
S. marcescens	SHV-7	-	-	neg	neg	0.5	1	<=0.125
Acinetobacter baumannii	IMP-1	+		pos	B	> 1	> 8	> 8
A. baumannii	IMP-4	+		pos	B	> 1	> 8	> 8
A. baumannii	NDM-1	+		pos	B	> 1	> 8	> 8
A. baumannii	NDM-1	+		pos	pos - untyped	> 1	> 8	> 8
A. baumannii	NDM-1	+		pos	B	> 1	> 8	> 8
A. baumannii	OXA-23, NDM	+		pos	D	> 1	> 8	> 8
A. baumannii	VIM-2	+		pos	pos - untyped	> 1	> 8	> 8
A. baumannii	VIM-2	-	+	pos	pos - untyped	> 1	> 8	> 8
A. baumannii	KPC	-	+	pos	D	> 1	> 8	> 8
A. baumannii	OXA 23	-	(w)	pos	D	> 1	> 8	> 8
A. baumannii	OXA-23	-	(w)	pos	D	> 1	> 8	> 8

Organism	Mechanism(s)	Rapidec 30 mins	Rapidec 2h	Phx Pos/Neg Result	Phx Classification	Phx ETP MIC	Phx IPM MIC	Phx MEM MIC
A. baumannii	OXA-23	-	+	pos	D	> 1	> 8	> 8
A. baumannii	OXA-23	-	+	pos	D	> 1	> 8	> 8
A. baumannii	OXA-23	-	+	pos	D	> 1	> 8	> 8
A. baumannii	OXA-23	-	+	pos	D	> 1	> 8	> 8
A. baumannii	OXA-23	-	+	pos	D	> 1	> 8	> 8
A. baumannii	OXA-23	-	+	pos	D	> 1	> 8	> 8
A. baumannii	OXA-23, OXA-40	-	(w)	pos	pos - untyped	> 1	> 8	> 8
A. baumannii	OXA-40	-	(w)	pos	pos - untyped	> 1	> 8	> 8
A. baumannii	OXA-40	-	+	pos	A	> 1	> 8	> 8
A. baumannii	OXA-40	-	+	pos	pos - untyped	> 1	> 8	> 8
A. baumannii	OXA-58	-	+	pos	B	> 1	> 8	8
A. baumannii	OXA 58	-	+	pos	D	> 1	> 8	8
A. baumannii	OXA-72	-	(w)	pos	D	> 1	> 8	> 8
Pseudomonas aeruginosa	IMP-1	+		pos	B	> 1	> 8	> 8
P. aeruginosa	IMP-7	+		pos	pos - untyped	> 1	> 8	> 8
P. aeruginosa	IMP-7	+		pos	B	> 1	> 8	> 8
P. aeruginosa	IMP-7	+		pos	B	> 1	> 8	> 8
P. aeruginosa	IMP-14	+		pos	pos - untyped	> 1	> 8	> 8
P. aeruginosa	IMP-18	+		pos	pos - untyped	> 1	> 8	> 8
P. aeruginosa	SPM	+		pos	pos - untyped	> 1	> 8	> 8
P. aeruginosa	SPM-1	+		pos	pos - untyped	> 1	> 8	> 8
P. aeruginosa	VIM	+		pos	B	> 1	> 8	> 8
P. aeruginosa	VIM	(w)	+	pos	B	> 1	> 8	> 8
P. aeruginosa	VIM	(w)	+	pos	B	> 1	> 8	> 8
P. aeruginosa	VIM	+		neg	pos - untyped	> 1	> 8	4
P. aeruginosa	VIM	+		neg	neg	> 1	> 8	4
P. aeruginosa	VIM-2	(w)	+	pos	B	> 1	> 8	> 8
P. aeruginosa	VIM-2	-	+	pos	B	> 1	> 8	> 8
P. aeruginosa	VIM-2	-	+	pos	B	> 1	> 8	> 8
P. aeruginosa	VIM-2	-	+	pos	B	> 1	> 8	> 8

Organism	Mechanism(s)	Rapidec 30 mins	Rapidec 2h	Phx Pos/Neg Result	Phx Classification	Phx ETP MIC	Phx IPM MIC	Phx MEM MIC
P. aeruginosa	VIM-2	-	+	pos	B	> 1	> 8	> 8
P. aeruginosa	VIM-2	-	+	pos	B	> 1	> 8	> 8
P. aeruginosa	VIM-2	+		pos	B	> 1	> 8	> 8
P. aeruginosa	VIM-2	-	+	neg	neg	> 1	> 8	4
P. aeruginosa	VIM-3	+		pos	B	> 1	> 8	> 8
P. aeruginosa	VIM-4	+		pos	B	> 1	> 8	> 8
P. aeruginosa	VIM-7, OXA-45	+		pos	pos - untyped	> 1	> 8	> 8
P. aeruginosa	KPC	+		pos	A	> 1	> 8	> 8
P. aeruginosa	KPC	+		pos	A	> 1	> 8	> 8
P. aeruginosa	KPC-5	+		pos	A	> 1	> 8	> 8
P. aeruginosa	KPC-5	+		pos	A	> 1	> 8	> 8

(w)*, borderline result

U*, uninterpretable test
